# Infectious Prions in Pre-Clinical Deer and Transmission of Chronic Wasting Disease Solely by Environmental Exposure

**DOI:** 10.1371/journal.pone.0005916

**Published:** 2009-06-16

**Authors:** Candace K. Mathiason, Sheila A. Hays, Jenny Powers, Jeanette Hayes-Klug, Julia Langenberg, Sallie J. Dahmes, David A. Osborn, Karl V. Miller, Robert J. Warren, Gary L. Mason, Edward A. Hoover

**Affiliations:** 1 Department of Microbiology, Immunology and Pathology, Colorado State University, Fort Collins, Colorado, United States of America; 2 National Park Service, Fort Collins, Colorado, United States of America; 3 Wisconsin Department of Natural Resources, Madison, Wisconsin, United States of America; 4 WASCO Inc., Monroe, Georgia, United States of America; 5 Warnell School of Forestry and Natural Resources, University of Georgia, Athens, Georgia, United States of America; Uppsala University, Sweden

## Abstract

Key to understanding the epidemiology and pathogenesis of prion diseases, including chronic wasting disease (CWD) of cervids, is determining the mode of transmission from one individual to another. We have previously reported that saliva and blood from CWD-infected deer contain sufficient infectious prions to transmit disease upon passage into naïve deer. Here we again use bioassays in deer to show that blood and saliva of pre-symptomatic deer contain infectious prions capable of infecting naïve deer and that naïve deer exposed only to environmental fomites from the suites of CWD-infected deer acquired CWD infection after a period of 15 months post initial exposure. These results help to further explain the basis for the facile transmission of CWD, highlight the complexities associated with CWD transmission among cervids in their natural environment, emphasize the potential utility of blood-based testing to detect pre-clinical CWD infection, and could augur similar transmission dynamics in other prion infections.

## Introduction

Chronic wasting disease (CWD) is a fatal transmissible spongiform encephalopathy (TSE), or prion disease, marked by a high transmission efficiency among cervids. Since its identification in cervid populations of northern Colorado and southeastern Wyoming [1]–[3], CWD has now been identified in 15 states, 2 Canadian provinces, and one Asian country [4]–[6]. Captive deer studies and epidemiological models of CWD prevalence and risk, suggest that the facile transmission of CWD between cervids occurs primarily by horizontal/lateral means [7]–[10]. CWD in farmed and free-ranging cervids has caused substantial economic, ecological, trade, and cultural impact and carries with it the potential for human and domestic animal health risk [11]–[20]. The latter, while at present theoretical, is shaped by the occurrence of human variant Creutzfelt Jacob Disease (vCJD) arising from consumption of bovine spongiform encephalopathy (BSE) [21]–[23] contaminated beef [24]. As with BSE, humans appear to be separated from CWD by a demonstrable species barrier [25], [26]. Nevertheless, the presence of infectious prions in the saliva and blood of terminal CWD-infected deer [27] provide a possible mechanism for natural transmission among cervids and provide an exposure risk to humans and domestic livestock. Additionally, demonstrated CWD infectivity in saliva and blood raises questions regarding the potential for horizontal transmission of other prion infections, especially during the subclinical or pre-symptomatic phase of disease as has been reported with blood transfusion of human vCJD [28]–[30], sheep scrapie [31], [32], and BSE infection [33]–[35].

We undertook the present studies to confirm our earlier findings on prion excretion through bioassays in white-tailed deer, but also to determine: (a) whether CWD prions are present in body fluids and excretions during the pre-clinical phase of infection, and (b) whether repeated environmental fomite exposure alone, vs. direct animal-to-animal contact, was sufficient to transmit CWD in deer.

## Results

### Detection of CWD infection in exposed animals

#### Blood (cohort 1)

**Figure 1:**
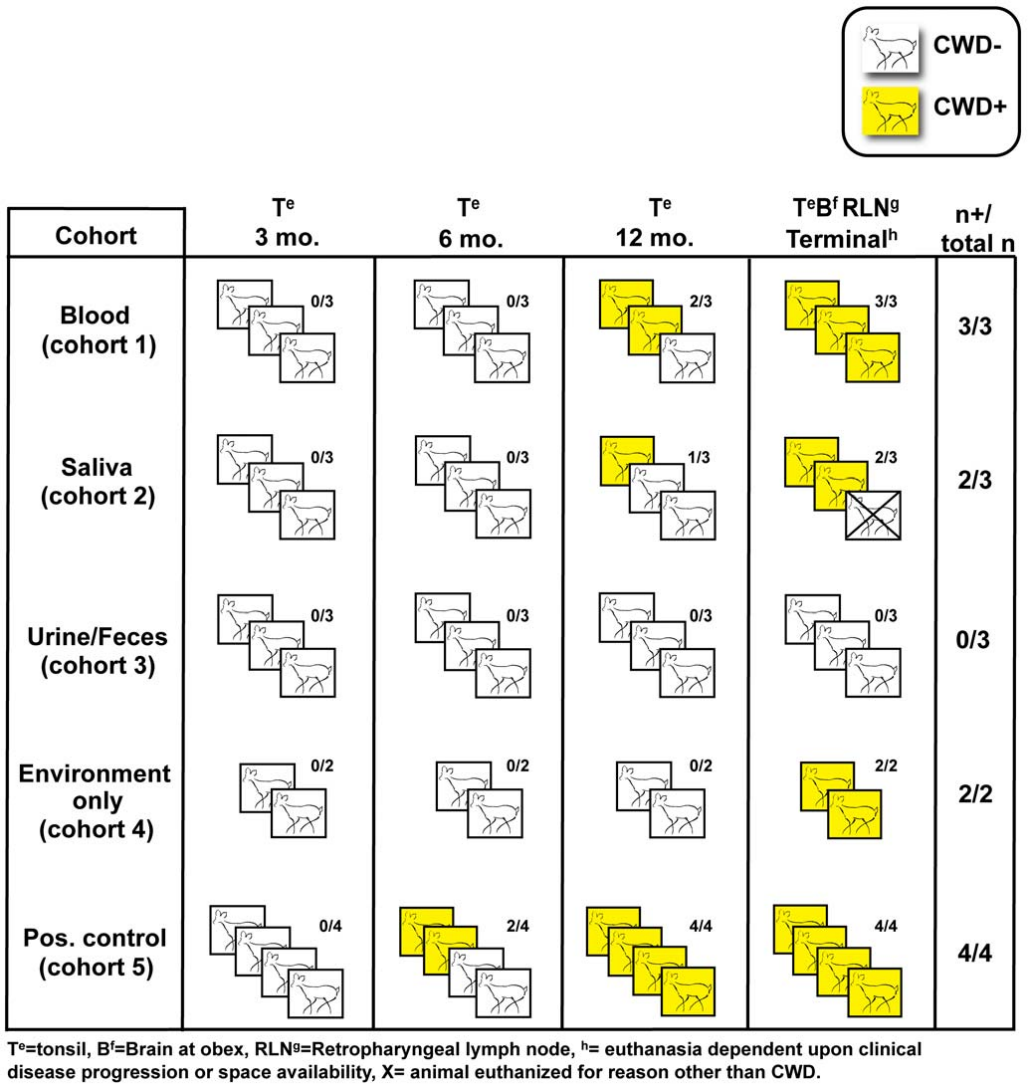
PrP^CWD^ detection by longitudinal tonsil biopsy and terminal necropsy.

**Figure 2:**
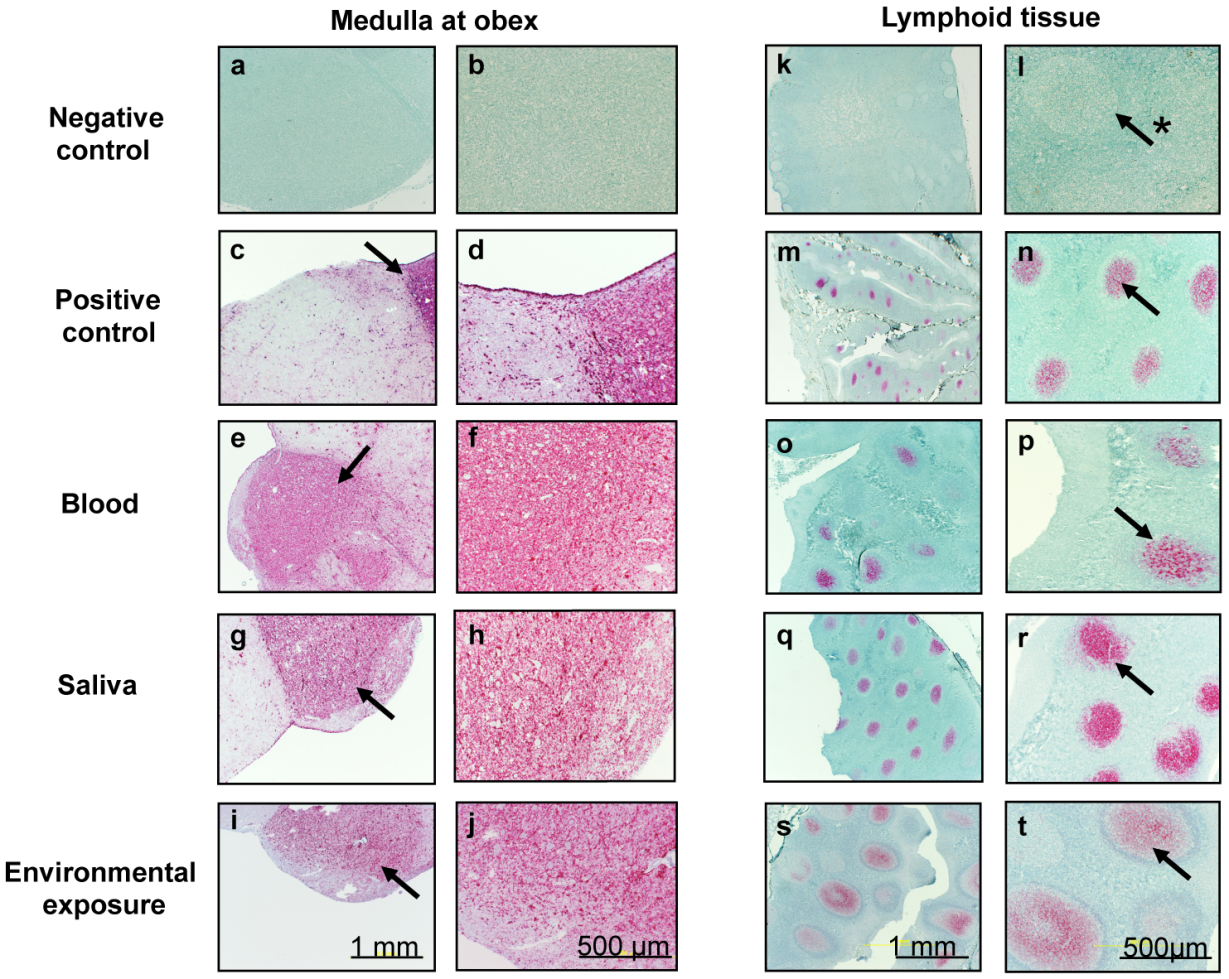
Immunohistochemistry results of deer exposed to body secretions and excreta from CWD+ deer. PrP^CWD^ demonstrated by immunohistochemistry in tonsil, brain (medulla oblongata at obex), and retropharyngeal lymph node of deer receiving saliva, blood, or environmental exposure from CWD-infected donors. CWD immunohistochemistry is shown in the medulla at obex (a–j) and either tonsil or retropharyngeal lymph node (k–t). Arrows indicate PrP^CWD^ staining (red) within brain and lymphoid follicles. Arrow with * indicates lymphoid follicle negative for PrP^CWD^.

Each naïve deer received blood by the IV route from pre-clinical CWD+ source deer that were 10–12 months post inoculation (pi) (Tables 1, 2). Two of three recipient deer became PrP^CWD^ tonsil biopsy positive at 12 months pi, but not at earlier sampling intervals (Fig. 1). At 19 months pi, when the cohort was necropsied, all three deer were CWD+, as indicated by detection of PrP^CWD^ in the medulla oblongata at the level of the obex (medulla at obex) and in lymphoid tissue (Fig. 2).

**Table 1:** CWD bioassay inoculation cohorts.

Animal cohort	*n*	Inoculum	Route of inoculation	Dosage (total volume)	No. of inoculations	*Prnp* codon 96 genotype *n* GG/*n* GS
1	3	Whole blood	IV	1 (225 ml)	1	2/1
2	3	Saliva	PO	10 ml/day for 5 days (50 ml)	5	3/0
3	3	Urine and Feces	PO	90 daily doses (85 ml+112.5 gr)	90	3/0
4	2	Environmental contact^a^	PO	19 mos. continuous exposure	Refreshed daily for 570 days (19 mos.)	1/1
5	4	Brain	PO	1 gr/day for 5 days (5 gr)	5	2/2

a Water, feed buckets and bedding from CWD+ deer suites.

**Table 2:** CWD bioassay inocula sources.

Animal cohort	Inoculum	Donor animal (n inoculated)	Donor animal clinical status	Donor animal mo. pi.
1	Whole blood	CSU Yr I 112^b^ (2)	Pre-clinical	10–12
		CSU Yr I 121^c^ (1)		
2	Saliva	CSU Yr I pool 112+121 (3)	Pre-clinical	6–13
3	Urine and feces	CSU Yr I pool 112+121 (3)	Transitioning^f^	3–24
4	Environmental contact^a^	CSU Yr I 121, 104, 106 (2)	Transitioning^f^	3–24
5	Brain	WDNR^d^ (2)	Mixed^g^	Terminal
		CSVDL TS 989^e^ (2)	Pre-clinical	

a Water, feed buckets and bedding from CWD+ deer suites.

b Colorado State University deer number 112 (PO brain inoculated deer from previous study[27]).

c Colorado State University deer number 121 (IC brain inoculated deer from previous study [27]).

d Wisconsin Department of Natural Resources, Madison, WI, USA.

e Colorado State Veterinary Diagnostic Laboratory, Fort Collins, CO, USA.

f Transitioning from pre-clinical to clinical.

g Contains brain tissue from deer of pre-clinical and clinical status at terminal field collection.

#### Saliva (cohort 2)

Each of the 3 deer in this cohort received saliva from pre-clinical CWD+ donors that were 6 to 13 months pi (Tables 1, 2). PrP^CWD^ was detected in tonsil of 1 of the 3 inoculated deer at 12 months pi, but not at earlier time points. By 19 months pi, study termination, a second animal was CWD+, by detection of PrP^CWD^ in brain and lymphoid tissue. The remaining deer was of necessity terminated at 16 months pi due to unmanageable aggressive behavior. This animal was CWD negative as determined by extensive western blot and IHC analysis (Figs. 1, 2).

#### Urine/feces (cohort 3)

Each of the 3 deer received repeated (90 daily) oral doses of urine and feces from CWD+ source deer ranging from pre-clinical to advanced clinical disease (Tables 1, 2). All of the recipient deer remained PrP^CWD^-negative throughout the 19-month study course (Table 1, Figs. 1, 2).

#### Environmental exposure (cohort 4)

The two animals in this cohort were exposed to daily introductions of feed buckets, water, and bedding removed from pens housing deer transitioning from pre-clinical to clinical phases of the disease (Table 1, 2). One of 2 exposed deer became tonsil biopsy PrP^CWD^-positive at 15 months pi. At study termination, 19 months pi, both animals were CWD+ (Figs. 1, 2).

#### Positive controls (cohort 5)

After oral inoculation with CWD+ brain homogenate, PrP^CWD^ was detected in the tonsil of 4 of 4 inoculated deer at either 6 (n = 2), or 12 months (n = 2) pi (Figs. 1, 2 and Tables 1, 2).

### Clinical signs of CWD

Project-dedicated caretakers intimately familiar with each animal observed the deer daily. Subtle clinical signs consistent with CWD were detected 12–20 months pi in 3 of 4 positive control animals (cohort 5), 2 of 3 animals receiving blood transfusion (cohort 1) and 1 of 3 deer orally inoculated with saliva (cohort 2). Clinical disease onset manifested primarily as perceived body muscle-mass reduction and gradual weight loss, which reached ≥20% of maximum body weight over 2 to 8 months. Other clinical signs included: rough hair coat due to piloerection and a body stance characterized by a low head position and wide leg stance. Changes in behavior included hyperphagia and polydipsia despite weight loss, and stereotypic movements including head tossing, repetitive and exaggerated lifting of the legs, diminished alertness, and occasionally aggressive behavior in the advanced stage of disease. Animals were euthanized when they displayed advanced clinical signs of CWD or at 19 months pi.

### Detection of PrP^CWD^ in tissues at necropsy

**Figure 3:**
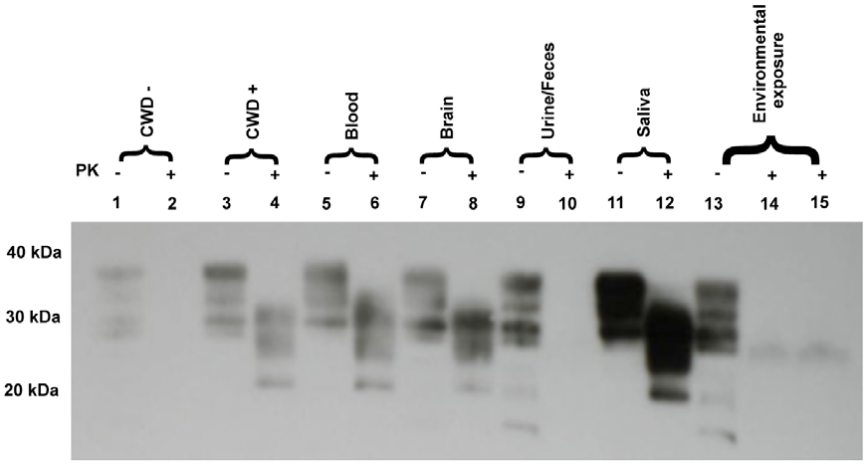
Western blot results of deer exposed to body secretions and excreta from CWD+ deer. Western blot demonstration of the typical PK digestion band shift (28–35 kD) associated with prion infection in the brain (medulla oblongata at obex) of deer receiving blood, brain, saliva or environmental exposure, but not urine and feces from CWD-infected donors. Lanes 1–4 represent CWD+/− deer controls (10% brain homogenate of medulla at obex) without and with PK digestion at 25 µg/ml. Lanes 5–12, 10% brain homogenate of blood, brain, urine/feces or saliva inoculated deer without and with PK digestion at 25 µg/ml. Lanes 13–15, brain homogenate from deer environmentally exposed to CWD+ fomites without and with PK digestion at 25 and 50 µg/ml.

Nineteen months after inoculation, all animals testing CWD− by tonsil biopsy in cohorts 1–4 were euthanized and necropsied. Two of 4 cohort 5 (positive control) deer and 2 of 3 cohort 1 (blood) tonsil-biopsy-positive deer were euthanized due to advanced clinical signs of CWD at 13, 24, 26 and 29 months pi, respectively. Earlier studies established that medulla at obex, tonsil and retropharyngeal lymph node had highest frequency of PrP^CWD^ deposition in infected deer [36]. Therefore, these tissues were rigorously examined in all animals. The results of immunohistochemistry (IHC) and western blot (WB) assays for PrP^CWD^ correlated with each other and with previous positive tonsil-biopsy results in 10 of 13 animals (Fig. 1). In 3 animals, however, (one each in the blood, saliva and positive control inoculation cohorts) in which previous tonsil biopsies had been negative, PrP^CWD^ was detected in tonsil, retropharyngeal lymphoid tissues and brain collected at necropsy (Fig. 1). Western blot analysis confirmed IHC results (Fig. 2) in all cases, and demonstrated the characteristic proteinase K-resistant 28–35 kd bands typical of PrP^CWD^ (Fig. 3).

## Discussion

### Time interval to detection of CWD infection by tonsil biopsy

**Figure 4:**
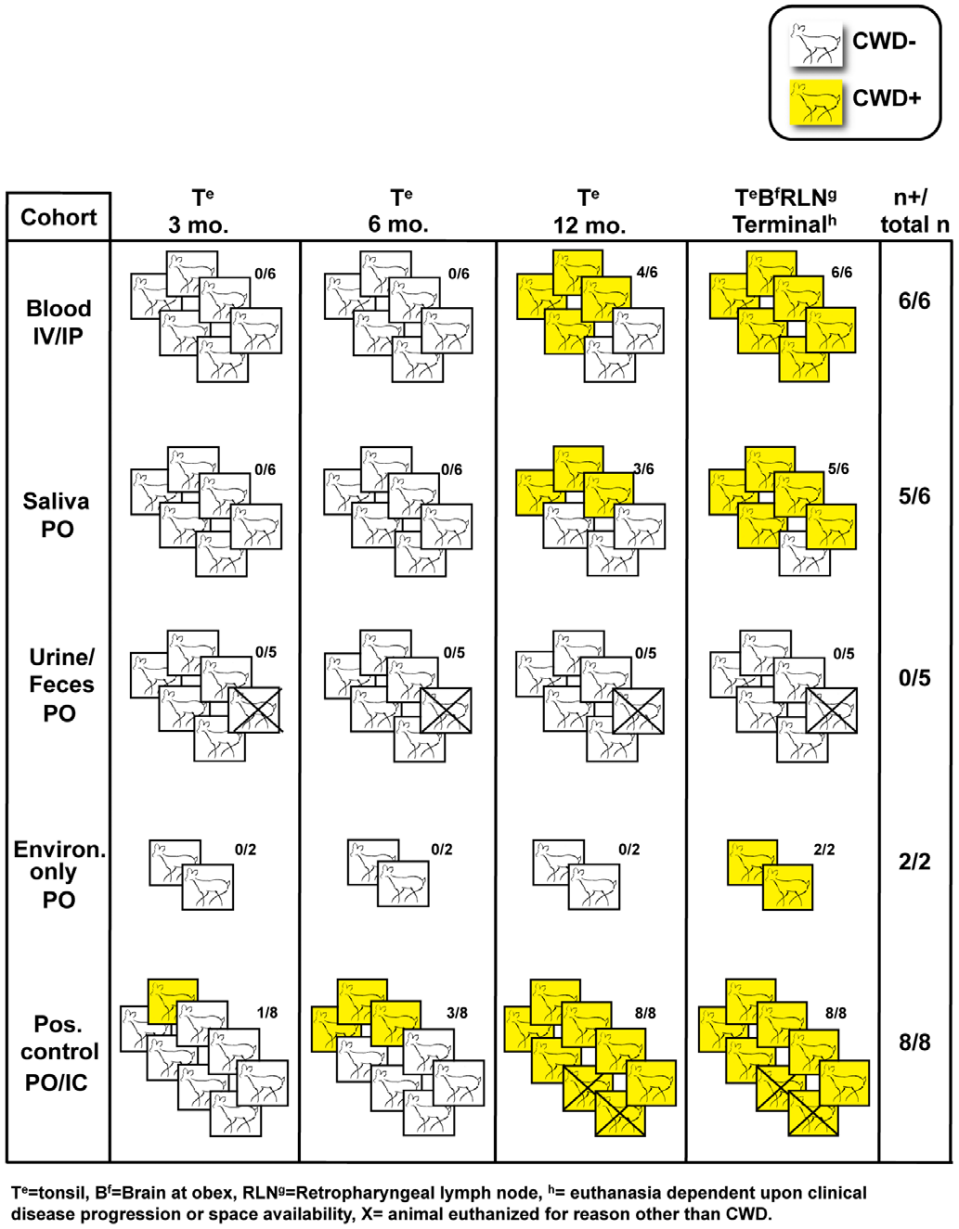
Summary of naïve deer exposed to inoculum from CWD+ deer—combined with our previous published findings [27].

The high transmission rate of CWD among cervids in their natural environment sets CWD apart from other prion diseases. The results of this study help provide a plausible basis for this facile transmission and extend our earlier findings [27] (Fig. 4) in demonstrating infectious prions in blood and saliva of pre-clinical CWD+ donors. The time from exposure to first detection of PrP^CWD^ by tonsil biopsy was variable—as short as 6 months but as long as 18 months. We assume that the time until appearance of PrP^CWD^ in tonsil is an underestimate due to the inherent variability in prion deposition kinetics [36] and the logistical limitations of tonsil biopsies, which require general anesthesia. The incubation periods prior to clinical CWD in our study were similar to those observed previously in experimental and naturally acquired infections [27], [37], [38]. While we can not exclude horizontal transmission from the first positive deer in each cohort, the timeframe for detection in the remaining deer (3 months) is less than half that which we have historically observed in deer inoculated orally with a brain homogenate from terminal CWD-infected deer [27], suggesting much earlier exposure to infectious prions.

### Infectious prions in saliva of CWD+ deer

The presence of infectious CWD prions in saliva suggests the potential for disease transmission via grooming interactions, shared water sources and communal feeding grounds—especially in high density cervid populations, such as those that exist for some cervid species during the breeding season, at baiting sites, in captivity, and in low predation situations. Other investigators have detected the presence of the aberrant misfolded prion protein (PrP^RES^) in alimentary tract tissue, and have suggested saliva as a possible vehicle for prion transmission [27], [37], [39], [40]. While the volume of saliva used in this study was large, the results nevertheless provide evidence to support the above premise. Salivary dissemination of prions may not be limited to CWD. Prions associated with transmissible mink encephalopathy (TME) have been detected in the submandibular salivary gland of mink [41] and TME protease-resistant prion protein has been detected in the lamina propria of the oral cavity, taste buds and squamous epithelium of the tongue, and the vomeronasal organ and olfactory mucosa of infected hamsters [40]. Hamster-adapted scrapie agent has been found in the tongue and taste buds of prion-infected hamsters [42]. Vascellari *et al* reported the presence of the pathological prion protein in both major and minor salivary glands of naturally and experimentally infected sheep [43], and we have made similar observations in the olfactory mucosa of ferrets experimentally infected with CWD [44] and in the taste buds of deer (Haley, NJ, personal communication). The exact source of prions shed in saliva remains speculative; possible sources include centrifugal/retrograde passage from nerve fiber terminations in the oral–nasal mucosa, or from lymphoid cells emanating from infected tonsilar or other alimentary lymphoid tissues.

### Infectious prions in blood of CWD+ deer

Blood-borne transmission of TSEs has long been feared, and the identification of a prion pathogen associated with blood-borne transmission has been pursued with disparate results [33], [45], [46]. Here we report the induction of CWD infection by a single blood transfusion from each of two pre-clinical CWD+ blood donors. This result is consistent with previous findings in substantiating the transmission of infectious prions by the blood of asymptomatic animal [27], [32] and human [28]–[30], [47], [48] donors, thus providing support for a subclinical hematogenous carrier state in TSE infections.

Direct detection of blood-borne PrP^RES^ has been difficult. Saa *et al* were the first to use protein misfolding cyclic amplification assay (PMCA) [49], [50] to detect protease-resistant prion protein in the blood of asymptomatic scrapie-infected hamsters [51]. More recently, Thorne *et al* reported PMCA amplification of PrP^SC^ from the blood of scrapie-infected sheep [52]. Continued efforts toward the development of sensitive, noninvasive, diagnostic tools are paramount. We are presently re-examining by serial PMCA the tissues of exposed but conventional PrP^CWD^ test negative animals that may harbor infectious prions not manifested in the observation periods used in our CWD studies.

Hunter and colleagues [33], [34] provided the first evidence for blood-borne TSE transmission for bovine spongiform encephalopathy (BSE) and scrapie by transfusion of whole blood [33], [34] and buffy-coat white blood cells [34] from infected donor sheep to naïve sheep. Sparse but compelling evidence has accumulated for blood transmission of variant Creutzfeldt-Jakob Disease (vCJD) [28]–[30], [48] and PrP^RES^ has been found in peripheral organs of some sporadic CJD patients [53], raising the possibility that peripheral distribution of PrP^RES^ is not limited to vCJD. In an ongoing study of sixty-six individuals who received blood products from asymptomatic blood donors who later developed vCJD [54], three of the 66 blood transfusion recipients developed vCJD 6.5 to 8.5 years after receiving blood [28], [30], [48] and a fourth blood recipient died of causes unrelated to vCJD five years after receiving the blood donation. Upon autopsy of this individual, PrP^RES^ was detected in lymphoid tissue but not brain, thus providing presumptive evidence for a case of subclinical infection [29]. Our findings with CWD further support the tenet that blood products from subclinical prion-infected individuals may transmit disease.

Additional cases of subclinical human prion disease may exist. While *in vitro* conversion studies have indicated an inefficient conversion of human PrP into a protease-resistant form [26], [55] and no evidence exists of CWD transmission to non-cervid species cohabitating with or on CWD contaminated environments [24], [56]–[58], it is reasonable to surmise that cross-species transmission of prions may require extenuating circumstances, i.e. origin of specific strains [59], [60], prolonged incubation time [61], and permissive genotypes [62]. At least two studies provide information bearing on these concerns. The first study, an ongoing longitudinal study to closely monitor 81 Americans who inadvertently consumed, or were exposed to, CWD+ venison at an upstate New York sportsman's feast, will conduct health evaluations of these individuals over the next six years [63]. The second, a retrospective study using western blot analysis of human tonsil and appendix samples collected in the United Kingdom (UK) to investigate possible exposure to the BSE agent, reported the detection of abnormal prion protein in three of 12,674 samples [64]. Mathematical modeling based on the results of this study predicts a minimum estimate of 3000 BSE infected people in the UK between 10–30 years of age. If this model is accurate, it predicts that 93% of these individuals could develop long-term subclinical infection [65].

### Environmental sources of CWD infection

Previous studies have confirmed direct animal-to-animal contact—horizontal transmission—as an efficient mode for prion disease transmission [9], [66]. Moreover, Miller and colleagues [9], [67], [68] have provided substantial evidence for environmental contamination as a source of CWD infection. Our bioassay study inocula doses (50 ml saliva/deer), while efficient in establishing the infectious nature of saliva, are likely unrealistic doses to be acquired in a natural setting. To emulate a more feasible natural environment-associated dose, while negating direct animal-to-animal contact, we exposed naïve deer to repeated exposures to fomites from the suites of CWD-infected deer. The study design was meant to mirror the daily habits and movements of a deer in its natural setting in which it may return to an area contaminated with small amounts of infectious prions over time. Here we provide the first report that under controlled indoor conditions CWD-naïve deer can acquire infection by exposure to fomites from the environment of CWD-infected deer, supporting the findings of Miller *et al* in the natural environment [9], [67], [68], in demonstrating that there are sufficient infectious prions in bedding and water to transmit CWD. Efficient transmission, as evidenced by tonsillar lymphoid PrP^CWD^ detection, was seen in as little as 15 months post initial exposure. These results are also consistent with the findings of Georgsson [69] and Miller [67] as part of their attempts to decontaminate areas heavily contaminated with scrapie and CWD. Animals reintroduced to these areas after decontamination developed clinical signs of prion disease within two years. The presence of infectious CWD prions in the environment therefore strongly suggests that natural prion infection occurs by routes additional to direct animal-to-animal contact. Based on the present and our previous findings [27], we speculate that saliva may harbor the greatest concentration of CWD prions available for horizontal transmission and environmental contamination, but recognize that other routes of excretion at lower concentration and greater volume still remain plausible.

### Lack of detectable infectious prions in the urine and feces of CWD+ deer

Previous studies have postulated that environmental contamination by excreta from infected cervids seems the most plausible explanation for the dissemination of CWD [70], yet at 19 months pi we were not able to detect PrP^CWD^ in the three deer inoculated with urine and feces. Our earlier report [27] indicated that 2 of 2 deer expressing the *prnp* gene G/S polymorphism at codon 96 remained negative 19 mo. pi. In the present study all three deer inoculated with urine and feces expressed the G/G polymorphism at *prnp* codon 96, which is associated with susceptibility to CWD infection [71]. We report no detection of PrP^CWD^ in the obex or lymphoid tissues of deer with either G/G or G/S polymorphisms at 19 mo pi. Although both of our bioassay studies in deer have failed to transmit CWD infection by oral exposure to urine and feces from CWD-infected deer, these results must still be interpreted with caution in light of ongoing PMCA and cervid transgenic mouse intracerebral bioassay studies which suggest that very low concentrations of prions may be present in urine and feces of CWD+ cervids [72]–[74]. Perhaps an incubation time longer than 19 months is necessary for a detectable accumulation of lymphoid PrP^CWD^, or a larger dose of inoculum by the oral route is necessary for efficient passage of prions across the alimentary mucosa.

In summary, the results reported here reconfirm that blood and saliva are sources of infectious CWD prions, consistent with previous findings [27], and further support a mechanism for efficient CWD transmission in nature. We also show that infectious prions shed into the environment by CWD+ deer are sufficient to transmit the disease to naïve deer in the absence of direct animal-to-animal contact. These observations reinforce the exposure risk associated with body fluids, excreta, and all tissues from CWD+ cervids and suggest that similar dynamics may exist in other prion infections.

## Materials and Methods

### Ethics Statement

All animals were handled in strict accordance with good animal practice as defined by relevant national and/or local animal welfare bodies, and all animal work was approved by Colorado State University Animal and Care Use Committee (ACUC approval number 08-175A-01).

### White-tailed deer

White-tailed deer fawns were provided by the Warnell School of Forestry and Natural Resources, University of Georgia, Athens (UGA)—a region where CWD has not been detected. The deer fawns were hand-raised and human and indoor-adapted before overnight transport directly to the Colorado State University (CSU) CWD research indoor isolation facility without contact with the native Colorado environment. The 4-month-old fawns were adapted to the facility housing conditions and diet for 2 months before study start.

### Genotyping

All white-tailed deer were genotyped to determine GG/GS (codon 96) status by the laboratory of Dr. Katherine O'Rourke, USDA-ARS, Pullman, WA.

### Biocontainment protocols

Protocols to preclude extraneous exposure and cross-contamination between cohorts of animals as previously described [27] incorporated protective shower-in requirements, Tyvek™ clothing, masks, head covers, and footwear, while maintaining stringent husbandry. Tonsil biopsy and terminal sample collections were taken with animal-specific biopsy and sample collection instruments to minimize possibility of cross contamination. Bedding and liquid waste from each suite was either incinerated or collected in a dedicated outdoor underground holding tank and denatured by alkaline digestion.

### Inoculation cohorts

Groups of n = 2 to 4 six-month-old fawns (Table 1) were housed in separate isolation suites throughout the study. Suite-dedicated protective clothing, utensils, and waste disposal were incorporated to exclude cross contamination by fomites, bedding, food, excretions, or contact.

### Inocula (route of inoculation; volume of inocula; months pi of donor animals)

The following inocula were used: Blood (intravenous- IV; 1 transfusion of 225 ml; 10–12 mo pi), saliva (oral- PO; 10 ml/day for 5 days = 50 ml total; 6–13 mo pi) were obtained from serial sample collections of pre-clinical CWD-inoculated, tonsil biopsy positive deer, while urine and feces (PO; 90 daily doses totaling 85 ml urine+112.5 gr feces; 3–24 mo pi) were obtained from serial sample collections of pre-clinical and clinical CWD-inoculated, tonsil biopsy positive deer housed in the CSU CWD isolation facility[27]. Positive control brain (PO; 1 gr/day for 5 days = 5 gr total; terminal field samples) homogenates (medulla oblongata at the level of the obex) that were confirmed as CWD+ by immunohistochemistry (IHC) were from one of two sources– either free-ranging white-tailed deer collected by the Wisconsin Department of Natural Resources (WDNR) (n = 2) or a free-ranging mule deer utilized in a previous bioassay study[27] provided by the Colorado State Veterinary Diagnostic Laboratory (CSVDL) (n = 2). For the environmental exposure study, water, feed buckets, and bedding were collected on a daily basis from CWD+ deer (3 to 24 months pi) that were part of ongoing studies in the CSU CWD research facility. The cohort of environmentally exposed deer was in uninterrupted contact with a continually refreshed supply of CWD+ fomites for 19 months (Table 2).

### Monitoring and sample collection

All animals were monitored for evidence of CWD infection by serial tonsil biopsies taken at 3, 6, 12 months pi, and at study termination. Tonsil tissue was divided and equal portions either stored at −70°C or fixed in 10% formalin for 24 hours before processing for IHC. At the same sampling intervals, blood, saliva, feces, and urine were collected from each animal and stored at −70°C. At necropsy, palatine tonsils, brainstem (medulla at the obex) and retropharyngeal lymph nodes, as well as other tissues, were collected for examination by IHC and western blotting (WB) to identify the presence of the protease-resistant prion protein associated with CWD (PrP^CWD^).

### Western blotting

Tissue homogenates were prepared from the obex region of the medulla oblongata encompassing the dorsal motor vagal nucleus (medulla at the obex). Twenty percent (20%) w/v homogenates were prepared in NP-40 buffer (10 mM Tris-HCl buffer pH 7.5, 0.5% NP-40, 0.5% sodium deoxycholate) by Fastprep™ disruption at setting 6.5 for 45 seconds. Twenty-five µl of each homogenate was mixed with 5 µl proteinase K (PK) (Invitrogen) to a final concentration of 25 µg/ml and incubated for 30 minutes at 37°C with shaking. Proteinase K activity was stopped with 4 µl 200 mM Pefablock SC and an equivalent volume of each sample was mixed with 10 µl sample buffer (Invitrogen−20% Reducing agent 10×, 50% LDS Sample buffer 4×), 5 µl NP-40 buffer (10 mM Tris-HCl pH 7.5, 0.5% deoxycholic acid, 0.5% nonylphenoxylpolyethoxylethanol), heated to 95°C for 5 minutes and separated by 12% Bis-Tris precast polyacrylamide gel electrophoresis (PAGE) (Invitrogen) at 150 volts for 2.5 hours in 1× MOPS (Invitrogen). PAGE separated proteins were transferred to polyvinylidene fluoride (PVDF) membrane for 1 hour at 100 volts in transfer buffer (0.025 M Trizma base, 0.2 M glycine, 20% methanol, pH 8.3). After the PVDF membranes were blocked overnight at room temperature in Pierce Blocker™ they were probed with the PrP specific antibody BAR224 (kindly supplied by Dr. J. Grassi) followed by horse radish peroxidase (HRP)-goat anti-mouse IgG diluted in Pierce Blocker™. Membranes were washed for 1 hour after blocking and between antibodies with wash buffer (0.1 M Tris, 0.15 M NaCl, 0.2% Tween 20 pH 8.0). To visualize PrP bands the PVDF membranes were developed with the Amersham™ ECL detection system and a digital GelDoc™ (Fuji Intelligent dark box) using LAS-3000 Lite ImageReader software.

*Immunohistochemistry* (IHC) was performed by employing protocols as described by Spraker *et al* [3]. Briefly, 3–5 mm sections of formalin fixed formic acid treated tissues were deparafinized at 60–70°C for 1 hour, rehydrated via a series of xylene/ethanol baths, and treated in formic acid a second time (5 minutes) prior to a 20 minute antigen retrieval (Dako Target Retrieval Solution 10×) cycle in a 2100 Retriever™ (PickCell Laboratories). Slides were further processed with the aid of a Ventana Discovery™ autostainer utilizing the Ventana Red Map™ stain kit, the PrP^CWD^ specific primary antibody BAR224 and a biotinylated secondary goat anti mouse antibody (Ventana). After autostaining, the slides were quickly rinsed in a warm water detergent solution, passed through a series of dehydration baths and cover-slipped.
